# Insomnia Symptoms and Associated Factors in Caregivers of Adult Hospitalized Patients

**DOI:** 10.3390/healthcare11060852

**Published:** 2023-03-14

**Authors:** Laura Fernández-Puerta, Germán Prados, María Dolores Quiñoz-Gallardo, Dolores Vellido-González, María Leticia González-Guerrero, Antonio Rivas-Campos, Eladio Jiménez-Mejías

**Affiliations:** 1 Department of Nursing, School of Health Sciences, University of Granada, 18071 Granada, Spain; 2 Virgen de las Nieves University Hospital, 18014 Granada, Spain; 3 Department of Preventive Medicine and Public Health, University of Granada, 18016 Granada, Spain

**Keywords:** sleep quality, caregivers, anxiety, sleep initiation and maintenance disorders

## Abstract

Caregivers experience high levels of emotional stress and must cope with several clinical and hospital-related environmental factors that seriously impact their night’s rest. The purpose of this study was to establish the prevalence of insomnia symptoms in a sample of caregivers of adult hospitalized patients and to examine the relationships between insomnia symptoms and patient and caregiver-associated factors. A total of 152 caregivers were enrolled from the two main hospitals in Granada, Spain. Sociodemographic, economic, and care-related data were collected. Insomnia symptoms, burden, anxiety and depression, social support, and resilience were assessed. Information on patients’ hospital admission, dependence, and neuropsychiatric symptoms was also obtained. Most caregivers were middle-aged women caring for their spouses. Self-reported insomnia prevalence was set at 45.4%. Comparison analyses between caregivers suffering from insomnia symptoms and non-insomniacs showed significantly higher burden, anxiety and depression and patients’ neuropsychiatric symptoms (*p* < 0.05) and lower resilience and social support in the former (*p* < 0.01). A regression analysis showed that anxiety (ORa = 1.15; *p* < 0.05) and higher caregiver education level (ORa = 5.50; *p* < 0.05) were factors significantly associated with insomnia symptoms. Patients’ neuropsychiatric symptoms showed a trend toward statistical significance as well (ORa = 1.09; *p* = 0.06). There is an acute need to address, prevent and treat insomnia problems in caregivers.

## 1. Introduction

Sleep quality results from a complex interplay of physiological, psychological and social factors [1]. Female sex, the aging process [2], and socioeconomic status [3] are some of the factors associated with a higher frequency of sleep problems. Similarly, caregivers have reported more sleep problems when compared to age-matched control non-caregiver adults [4]. The most frequent complaints of this population include difficulties falling asleep, getting little sleep because of the continuous interruptions to provide care, and insomnia [4,5,6]. These sleep-related problems have been linked to caregiver burden, fatigue, and anxiety and depression symptoms [5,7]. Therefore, they may hamper or limit the caregiver role [8]. In fact, it has been reported that sleep problems among caregivers of patients with dementia are the first cause of admission of care recipients into a nursing home [9]. As regards insomnia, a high prevalence has been observed in family caregivers of care recipients with dementia and cancer [10,11]. Suffering from chronic insomnia has been related to serious consequences for physical and mental health; this increases the morbidity and mortality of people who experience sleep deprivation [12].

According to the model on the development of chronic sleep disorders in caregivers and care recipients by McCurry et al. [7], the development of insomnia or other types of alterations in sleep quality among caregivers can be determined by several factors related to their own physiological characteristics, life events, and behavioral and environmental factors. In addition to these risk factors linked to caregivers, there are factors associated with care recipients (e.g., age, dementia, recipient care needs) that can lead caregivers to have poor quality sleep in a dyadic relationship [7].

Care recipient hospitalization is a stressful event for caregivers, causing them concern and uncertainty about the health of their loved ones [13]. Due to this, hospitalization can behave as a precipitating or perpetuating factor for insomnia. It is estimated that 36–50% of hospitalized patients suffer from insomnia [14]. These sleep problems are closely related to higher morbidity and mortality, increasing the length of hospital stay [15]. However, few data are available regarding caregiver sleep quality during hospitalization. A study on caregivers of patients admitted to the intensive care unit (ICU) showed that up to 58% of caregivers suffer from sleep problems assessed by the General Sleep Disturbance Scale (GSDS) [16]. Outside ICU, caregivers’ experiences and sleep quality have only been addressed in pediatric wards [17]. These studies found caregivers to have poor sleep quality while staying overnight at the hospital [18,19]. However, the characteristics of caregivers of adults in general wards greatly differ from those of ICU and pediatric caregivers.

Therefore, although sleep problems in community-dwelling caregivers are common and have serious consequences on caregivers’ health and care recipient care, to date, few studies have addressed sleep troubles or their related characteristics during adult care recipient hospitalization.

### Aims and Objectives

The purposes of this study were: (1) to determine the prevalence of insomnia symptoms in a sample of informal caregivers of adult hospitalized care recipients (ICHPs); and (2) to identify and quantify the strength of association between insomnia symptoms and other emotional health variables, caregiver burden, and care-related variables.

## 2. Method

### 2.1. Study Design

The present study was a descriptive observational study of the retrospective case series type with a sample of caregivers looking after an adult family member during hospitalization. Data were collected from January 2017 to March 2020 via individual interviews. The STROBE statement was used to guide the reporting of this study [20].

### 2.2. Participants

Data were collected from January 2017 until just before the pandemic situation in our country (March 2020) via individual interviews. Given that one of the future objectives of this line of research is to build a cohort of informal caregivers, sample size was not previously predetermined and as many caregivers as met the inclusion criteria were included in the study. A total of n = 152 caregivers were evaluated. The study took place in Virgen de las Nieves University Hospital and San Cecilio University Hospital (Granada, Spain). Under the functional plan of the two main hospitals of the city for providing support to informal caregivers, inclusion criteria included: (i) being over 18 years and under 85 years old; (ii) being able to speak Spanish; (iii) providing care to a family member at the hospital; and (iv) meeting hospital criteria to become an ICHP [21]. Exclusion criteria included: (i) serious medical conditions which would hamper the provision of care or pregnancy; (ii) suspected or diagnosed sleep disorders; (iii) serious psychological problems such as psychotic disorders, major depression, suicidal ideation, or substance abuse; (iv) financial remuneration from the patient or other member of the family for the care provided; (v) hospital discharge in the three coming days from the start of the evaluation.

### 2.3. Measures

- Insomnia symptoms/Insomnia Severity Index (ISI) [22,23]. This questionnaire provides a global measure of the patient’s perception of insomnia symptoms severity and its impact on daytime functioning. It comprises five Likert scale items where higher scores indicate greater insomnia symptoms. Four severity groups can be distinguished according to scores: absence of clinical insomnia (0 to 7 points), subclinical insomnia (8 to 14 points), moderate insomnia (15 to 21 points), and severe insomnia (from 22 to 28 points).
- Caregiver burden/Caregiver Burden Interview (CBI) [24,25]. It includes twenty-two Likert scale items ranging from 1 “never” to 5 “always”. Scores greater than 55 indicate severe burden, whereas scores lower than 46 indicate no burden.
- Anxiety and depression/Hospital Anxiety and Depression Scale (HADS) [26,27]. It contains fourteen multiple-choice questions with anxiety and depression subscales. Subscale scores greater than 11 indicate probable cases.
- Patient dependence/Barthel Index [28,29]. It is a fifteen-item questionnaire that assesses impairment in daily living activities. Higher scores indicate greater independence (<20 total dependence, 21–60 severe dependence, 61–90 moderate dependence, and 91 or more minor dependence).
- Patient neuropsychiatric symptoms/Neuropsychiatric Inventory Questionnaire (NPI-Q) [30,31]. This is a twelve-item questionnaire for neuropsychiatric symptom evaluation (i.e., hallucinations, delusions, agitation/aggression, dysphoria/depression, anxiety, euphoria/elation, apathy/indifference, disinhibition, irritability/lability, aberrant motor behavior, nighttime behavioral disturbances, and appetite/eating disturbances). Caregivers report the frequency and intensity of symptoms. Higher scores indicate higher severity (range 0–36).
- Social support satisfaction/Social Support Questionnaire-Short Form (SSQ6) [32,33]. It includes six items in which participants quantify the availability and satisfaction from perceived social support. In this study, we used the satisfaction subscale, whose scores range from 1 to 6 (very dissatisfied to very satisfied).
- Resilience/Connor–Davidson Resilience Scale (CD-RISC) [34,35]. It comprises twenty-five Likert scale items from 0 (not true at all) to 4 (true nearly all the time). Results range from 0 to 100, with higher scores indicating higher resilience.

Other variables included age, gender, the relation between caregiver and care recipient, education level, job status, caregiver’s regular medication, caregiving time, and sleeping site. Age, gender, hospitalization unit, and days spent since hospital admission were recorded from patients’ clinical records.

### 2.4. Data Collection

**Figure 1:**
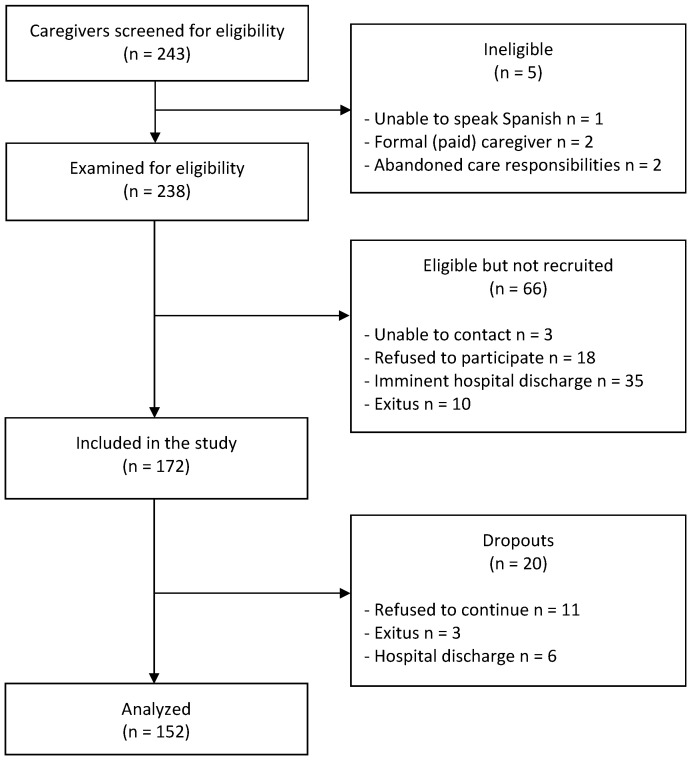
Flow diagram.

All eligible individuals were invited to participate in a study aimed at assessing if the care provided by caregivers had an impact on their sleep, mood, or other aspects during care recipient hospitalization. Social-health data were collected from hospital clinical records. Consecutive sampling was used to obtain the highest possible representativeness of the sample (see Figure 1).

Individuals who accepted to participate in the study were cited for an individual semi-structured interview. Sociodemographic, socioeconomic, caregiver health, and care-recipient disease data were assessed. Next, caregivers received a battery of validated self-reported questionnaires. After 7 days (or before that because of patients’ discharge), participants were cited again and questionnaires were collected. Investigators were available during these days to support caregivers who had inadequate reading skills or other problems in filling out self-reported measures.

### 2.5. Ethical Considerations

This study was approved by the by the Ethics Committee of Granada Province (reference code: 1451-N-17.I.P.). All participants received information regarding study goals and signed a prior informed consent form. To ensure the confidentiality of the data, participants were anonymized through a number that only the researchers could access. Informed consent and personal data were kept separately.

### 2.6. Data Analysis

Quantitative data were expressed through means and standard deviations, while qualitative data were expressed through frequencies and percentages. To determine the prevalence of insomnia symptoms, clinical cut-off values from the ISI were used [22]. Two large groups were established: caregivers without insomnia symptoms (ISI scores ranging from 0 to 14) and caregivers with insomnia symptoms (ISI scores ranging from 15 to 28). Next, Chi-square, Fisher’s Exact Test and Student’s *t*-tests were used to determine raw associations between insomnia symptoms and other variables. Finally, to estimate the association between being an ICHP and the presence of insomnia symptoms, the corresponding crude and adjusted ORs were estimated for the main potentially confounding factors: sex and age, education level, hospital stay hours and sleeping site, hypnotics intake, burden, anxiety and depression, patient’s Barthel and neuropsychiatric symptoms, social support, and resilience. Logistic regression models were performed for this purpose. All analyses were carried out using Stata 16.0 (Stata Corp. LLC, College Station, TX, USA).

## 3. Results

A total of n = 152 ICHPs completed the evaluation. The average age was 55.88 ± 12.81 years (range 26–80). Participants were mostly married (80.3%), female (83.6%), with a basic-medium education level (82.3%), and were caring for their spouse or a parent (80.3%). The mean time of caregiving was 4 years (range 15 days–43 years) and participants cared for the family member at the hospital for more than 5 days a week. Regarding caregivers’ resting place while their loved ones were hospitalized, 57.2% of caregivers spent the night in the hospital and most of them rested in a recliner chair at the patient’s bedside (86.6%). In addition, 32.9% of the whole sample used hypnotics to sleep (see Table 1).

Regarding patient characteristics, the average age was 61.38 ± 18.54 years (range 14–90). In most cases, patients were admitted to medical units (75%), with 32.65 ± 35.47 days since admission (range 2 days–7 months) at the beginning of the interview (see Table 2). Most patients (86%) had moderate to severe dependence.

The prevalence of insomnia symptoms in ICHPs was set at 45.4% (n = 69) using the ISI questionnaire. Caregivers with insomnia symptoms scored an average of 18.72 ± 3.38 points in the ISI, while those without insomnia scored 9.23 ± 3.61 points. Group comparisons (ICHPs suffering from insomnia symptoms vs. not suffering from insomnia symptoms) showed no significant differences in sociodemographic or care-related variables related to hospitalization, excluding education level (*p* < 0.05). We found that ICHPs suffering from insomnia symptoms showed higher levels in burden, depression, and anxiety and a worse perceived social support and capacity of resilience as compared to those without insomnia (see Table 1). At the same time, care recipients of ICHPs with insomnia symptoms showed more neuropsychiatric symptoms (see Table 2).

Table 3 shows the estimates of crude and adjusted associations between insomnia symptoms and being a caregiver using logistic regression models. Despite the modest size of our sample, this table highlights that the variables most strongly associated with insomnia symptoms in ICHPs were having a high education level, with an ORa of 5.50 (95% CI: 1.34–22.63) and having anxiety symptoms, with an ORa of 1.15 (95% CI: 1.01–1.31) (*p* < 0.05). In the crude model, all the psychological variables together with social support and patients’ symptoms were also linked to this variable and statistical significance was even reached. Other variables that seemed related to insomnia symptoms, although no statistical difference was reached, were medium education level (ORa = 1.16; 95% CI: 0.36–3.73) and patients’ severity of neuropsychiatric symptoms (ORa = 1.09; 95% CI: 1.00–1.20) (*p* = 0.06). By contrast, variables associated with a lower frequency of insomnia were caregiver female sex (ORa = 0.86; 95% CI: 0.23–3.18), not taking hypnotics (ORa = 0.53; 95% CI: 0.20–1.45), sleeping at home (ORa 0.65, 95% CI: 0.12–3.65) or combining rest at home with the hospital (ORa = 0.85; 95% CI: 0.22–3.31), and a higher level of social support satisfaction (ORa = 0.79; 95% CI: 0.55–1.13).

## 4. Discussion

A sudden change in the care recipient’s health and consequent hospitalization are stressful events that seriously impact caregivers’ health and burden. This situation may worsen if the caregivers stay at their care recipient’s bedside overnight. Specifically, sleep disturbances in caregivers can develop or worsen during admission, mediated by psychological, environmental, and care-related factors. Few studies have addressed this phenomenology among ICHPs of adult patients during hospitalization. Consequently, the present study aimed to examine the prevalence of insomnia symptoms and their associated risk factors in different hospital units in a tertiary hospital complex.

Our analyses revealed that almost half of ICHPs had clinical insomnia symptoms (45.4%), which is significantly higher than insomnia complaints in healthy middle-aged adults in our country (29.1%) [36]. This finding is in line with the findings of other authors, who estimate the prevalence of sleep disturbances at 50–74% and at 40–76% for community-dwelling caregivers of care recipients suffering from dementia and cancer, respectively [10,11]. Considering insomnia, Morris et al. [37] found that 30% of caregivers showed insomnia symptoms when using the same ISI cut-off. Specifically in the hospital setting, up to 66% of ICU caregivers and 59% of neurosurgical caregivers, respectively, reported having difficulty sleeping [16,38]. Poor sleep was also experienced in pediatric wards due to anxiety, environmental noise, and child-related factors [19]. However, a comparison of our results with those of previous studies should be considered with caution. First, caregiver characteristics in our study are heterogenous in terms of care recipient diagnosis, age, and dependence level. Second, most of the studies assessed sleep disturbances using instruments that are not specifically designed for the assessment of clinical insomnia (e.g., the Pittsburgh Sleep Quality Index). Third, there is no previous research about sleep in ICHPs of adult patients in general hospital wards when they stay overnight in the hospital. Thus, comparisons with previous studies should take into account the complexity of factors acting in the hospital environment as compared to community dwelling [39].

Of the 152 caregivers in our study, 83.6% were women caring for their spouse or a parent (80.3%). From an epidemiological point of view, we can consider that the sociodemographic features of our sample are in agreement with the caregiver general population [40]. Although no significance was reached, in our study we found that the female sex was associated with a lower frequency of having insomnia symptoms. This fact differs from the available literature, according to which female caregivers [5] and non-caregivers [2] report more sleep problems than males. This may be due to factors such as sexual hormones or stress [41,42]. Indeed, perceived stress has been found to be a predictor of insomnia for female caregivers, but not for male caregivers [43]. In addition, in our study age did not seem to be related to insomnia symptoms. This disagreement between our results and previous literature may be explained in part because the present study analyzed a practically unexplored context of informal caring (i.e., during patient admission) in a sample of middle-aged caregivers. Previous research about sleep troubles among informal caregivers has mainly been conducted in community-dwelling care recipients, a context in which female informal caregivers are the main people responsible for providing care to their loved ones. Acute hospitalization can be a stressful event that may hamper sleep, as we stated above. However, older women may feel some relief from their caring duties and burden overall when they have provided care for a long time, after a situation that probably worsened in the last weeks because of the acute illness of the care recipient. Beyond this conjecture, partly derived from the authors’ clinical experience, future research is warranted to corroborate our findings in this regard.

Education level seems to have an important impact on ICHP rest. Caregivers with a high education level had five times more insomnia complaints than those with a basic education level (*p* < 0.05). To a lesser extent, caregivers with a medium education level seem to suffer from this increased risk as well. This important finding may be in line with the fact that these individuals probably combine their role as a caregiver with another job and have compartmental patterns that are different from the traditional role of women exclusively dedicated to care. Similarly, Al-Zahrani et al. [44] found that well-educated caregivers were identified as being at higher risk of developing stress, anxiety, and depression during the hospitalization of their care recipients. Although that study did not focus on sleep, this also suggests that higher-educated caregivers experience more difficulties when caring for someone in the hospital setting.

Sleeping site has been found to be an important characteristic in our study. Only 19.1% of caregivers rested at home during care recipient admission to hospital and another 23.7% alternated home rest with hospital rest. Other studies have found different percentages from those referred to here, but important characteristics differed. During care recipient admission to the intensive care unit, 27% of caregivers rested in the waiting room, while the others did it at home or at a hotel [16]. Caregivers who rested at least one night in the hospital reported more sleep disturbances, anxiety, and fatigue than those who never slept overnight in the hospital. They concluded by stating that available hospital accommodations for family members are inadequate for sleeping. Although results were not significant, our study also found a small protective effect of sleeping at home. During hospitalization, there are both intrinsic (i.e., patients’) and extrinsic factors that can contribute to developing sleep problems in caregivers. Extrinsic factors include noise, light, changes in nighttime sleep habits, shared rooms, monitor alarms, medical tests, or vital sign measures, among others [15]. As a result, caregivers should be encouraged to rest at home. Healthcare workers should also take into account caregiver sleep, relieving caregivers from care recipient needs during hospitalization [45].

In the present study, a high proportion of caregivers took hypnotics (32.9%). Some studies described a low use of hypnotics in caregivers because of their sedative effect [6], but other studies have noted that the intake of hypnotics is higher than expected and can reach up to 22.3% [46]. Chronic pharmacological treatment has been related to serious adverse events. In addition, there is not sufficient empirical evidence of its effectiveness without any other sleep treatment [47]. Therefore, it is important to identify and propose preventive strategies for caregivers at risk of chronic consumption of hypnotics.

Regarding the psychosocial variables in our study, ICHPs with insomnia symptoms scored higher in anxiety, depression, and burden (*p* < 0.01), and lower in resilience and social support (*p* < 0.05) as compared to those without insomnia symptoms. Yet, only anxiety was a psychological predictor of insomnia symptoms (*p* < 0.05), although social support could act as a protector factor as well (Ora = 0.79; 95% CI: 0.55–1.13). ICHPs score high in depression, anxiety, stress [44], and burden [48], and low in social support [49]. A study on caregivers of patients admitted to the ICU showed that younger caregivers and the presence of stress were predictors for sleep quality assessed by GSDS, whereas social support was not associated [50]. However, in this study caregivers mostly rested at home. Moreover, other psychological factors such as anxiety, depression, or burden were not assessed. In community-dwelling caregivers, insomnia symptoms are associated with caregiver anxiety and depression [51]. Further research is needed to better understand how sleep is related to psychosocial variables and determine the impact of it on caregiver wellbeing.

Concerning patient characteristics, we found that neuropsychiatric symptoms were worse among care recipients whose caregivers complained of insomnia symptoms (*p* < 0.05). In addition, the regression analysis showed a trend toward statistical significance in increasing the risk of developing insomnia when those clinical symptoms increased in the patient, but no differences were seen regarding patients’ dependence (*p* > 0.05). The available literature about sleep in caregivers focuses on behavioral disturbances as the main disruptive characteristic of patients. One study found that neuropsychiatric symptoms could be a predictive factor of sleep disturbances in caregivers of persons with dementia [52]. Moreover, previous studies have described that patients’ lower physical function is associated with caregiver anxiety [53] and burden [54], but not directly with sleep. However, as this study was carried out in the hospital setting, it is reasonable to infer that neuropsychiatric symptoms have a greater influence on insomnia symptoms than patient dependence does since all patients’ needs were met by the nursing team.

Given the prevalence of insomnia symptoms and their associated factors in ICHPs, interventions are urgent to implement. There is evidence that cognitive-behavioral interventions, caregiver health interventions, and exercise programs have beneficial effects on caregiver quality of sleep [55]. However, interventions should not only address the caregiver itself, but also the hospital environment. Some studies have developed sleep-promotion protocols for reducing noise and other sleep disruptors in the hospital, improving patients’ sleep quality and reducing noise levels [56,57]. Future interventions in the hospital should involve caregivers as a key part to pursue greater and longer-term benefits in the dyad.

### Limitations

It should be noted that this is a descriptive study of a retrospective case series without a control group held in some hospitals of a given city. This, without a doubt, makes it impossible to verify any type of causal hypotheses, and our results only provide certain empirical evidence of the associations identified. Thus, while it is true that results are consistent with the available literature, we cannot generalize them to all caregivers of hospitalized patients. What is more, the social and cultural context plays an important part in this. Care-related characteristics and hospital policies regarding accompaniment significantly differ. Exceptional circumstances due to COVID-19 have recently changed all hospital policies, and these changes will probably persist indefinitely (even though this study was conducted before COVID-19). As a result, the generalizability of the results may be limited.

Due to our study design, we ignore whether caregivers had insomnia symptoms before patients’ hospital admission or whether hospital admission contributed to its development. However, we know that hospitalization can increase the problem. We also ignore the evolution of the outcome after hospital discharge.

Second, it is important to highlight the sample size and high rejection rates during the study. We had to stop the evaluations due to COVID-19. However, the fact that one of the long-term objectives of this pilot study was the creation of a cohort of caregivers supports the idea of conducting it even with a small sample size that did not make it possible to reach the level of significance. However, there are clear trends in the point estimates. In addition, many caregivers complained about “lack of time”. To all this, it is necessary to add dropouts due to patient worsening or exitus. As a result, we cannot guarantee that these caregiver characteristics are the same as those of the caregivers who completed the evaluation. Great caution should be taken in this regard.

Another important limitation is that we did not assess patients’ sleep quality, even though studies have found a dyadic relationship with caregiver sleep quality [8]. Moreover, because of the natural complexity of sleep, it is recommended to record it with both subjective and objective measures [9]. Polysomnography is considered the gold standard for sleep evaluation [58], but other devices such as actigraphy are reliable instruments for obtaining sleep information in a cheaper and easier way [59,60]. However, perceptions of sleep are only described using subjective measures. Other subjective assessments, such as the Pittsburgh Sleep Quality Index, shed more light on caregiver rest, providing information on sleep quality, sleep latency, and sleep efficiency, for example. Yet, questions referred to the past month, so we were not able to use this index in this study (because of recent hospital admission). Additionally, although the ISI is a reliable and valid instrument to detect patients with insomnia [22], meeting the diagnostic criteria for insomnia requires a clinical evaluation. As a result, the measures obtained by the ISI may have overestimated the prevalence of clinical symptoms of insomnia in our study.

Despite this, the fact of having identified the main confounding variables, the use of estimates adjusted for such variables, and the consistency of our results with those of other studies, are arguments in favor of the validity of our research. Further studies aiming to analyze caregiver sleep quality during care-recipient hospitalization are needed to confirm these findings. In addition, further analysis and examination are necessary to determine the internal and external characteristics related to insomnia during hospitalization.

## 5. Conclusions

Almost half of caregivers have insomnia during adult patient hospitalization and it is predicted by anxiety symptoms, higher caregiver education level, and patients’ neuropsychiatric symptoms. Insomnia is also associated with higher caregiver burden, anxiety, depression, and patient neuropsychiatric symptoms and lower caregiver resilience and social support. Knowing that hospitalization is an acute situation that can result in a chronic sleep disorder and taking advantage of the proximity to the healthcare team during admission, hospitalization could be a good time to develop effective sleep interventions for this population.

## Figures and Tables

**Table 1:** Distribution of the baseline characteristics of the sample of informal caregivers according to the presence of insomnia symptoms.

	Total (n = 152)		Insomnia Symptoms (n = 69)		No Insomnia Symptoms (n = 83)		
Characteristics	Mean (SD)	n (%)	Mean (SD)	n (%)	Mean (SD)	n (%)	*p* Value
**Age**	55.88 ± 12.81	-	54.41 ± 11.07	-	57.11 ± 14.04	-	0.196
**Sex**	-		-		-		0.141
Male		25 (16.4)		8 (11.6)		17 (20.5)	
Female		127 (83.6)		61 (88.4)		66 (79.5)	
**Education level**	-		-		-		0.016 *
Basic		79 (52.0)		31 (44.9)		48 (57.8)	
Medium		46 (30.3)		19 (27.5)		27 (32.5)	
High		27 (17.8)		19 (27.5)		8 (9.6)	
**Job status**	-		-		-		0.193
Employed		29 (19.1)		16 (23.2)		13 (15.7)	
Unemployed		53 (34.9)		22 (31.9)		31 (37.3)	
Retired		40 (26.3)		14 (20.3)		26 (31.3)	
Other		30 (19.7)		17 (24.6)		13 (15.7)	
**Caring for:**	-		-		-		0.918
Parent		33 (21.7)		14 (20.3)		19 (22.9)	
Son/daughter		17 (11.2)		8 (11.6)		9 (10.8)	
Spouse		89 (58.6)		42 (60.9)		47 (56.6)	
Other family member		13 (8.6)		5 (7.2)		8 (9.6)	
**Hospital stay** (hours per week)	130.27 ± 45.14	-	132.93 ± 43.95	-	128.06 ± 46.26	-	0.510
**Sleeping site**	-		-		-		0.420
Hospital ^a^		87 (57.2)		42 (60.9)		45 (54.2)	
Home		29 (19.1)		10 (14.5)		19 (22.9)	
Both		36 (23.7)		17 (24.6)		19 (22.9)	
**Hospital sleeping site**	-		-		-		0.987
Recliner		110 (86.6)		52 (86.7)		58 (86.6)	
Other		17 (13.4)		8 (13.3)		9 (13.4)	
**Time caring** (months)	47.11 ± 78.76	-	42.45 ± 53.54	-	51.03 ± 95.10	-	0.509
**Intake of hypnotics**	-	50 (32.9)	-	28 (40.6)	-	22 (26.5)	0.066
**ISI**	13.54 ± 5.89	-	18.72 ± 3.38	-	9.23 ± 3.61	-	-
**ZBI**	50.24 ± 13.44	-	55.55 ± 14.37	-	45.77 ± 10.82	-	<0.001 **
**HADS Depression**	9.41 ± 4.69	-	11.42 ± 4.51	-	7.72 ± 4.16	-	<0.001 **
**HADS Anxiety**	10.85 ± 4.80	-	13.14 ± 4.38	-	8.93 ± 4.28	-	<0.001 **
**SSQ6 Satisfaction**	4.74 ± 1.27	-	4.49 ± 1.30	-	4.95 ± 1.22	-	0.028 *
**CD-RISC**	70.93 ± 15.08	-	68.11 ± 15.76	-	73.32 ± 14.13	-	0.038 *

^a^ At least 80% of days since admission. * *p* < 0.05; ** *p* < 0.01. Continuous variables were analyzed by an Independent Samples *t*-test, and qualitative variables by Fisher’s Exact Test and Pearson Chi-Square. CD-RISC: Connor–Davidson Resilience Scale; HADS: Hospital Anxiety and Depression Scale; ISI: Insomnia Severity Index; SD: standard deviation; SSQ6: Social Support Questionnaire-Short Form; ZBI: Zarit Burden Interview.

**Table 2:** Distribution of patient characteristics according to the presence of caregivers’ insomnia symptoms.

	Total (n = 152)		Insomnia (n = 69)		No Insomnia (n = 83)		
Characteristics	Mean (SD)	n (%)	Mean (SD)	n (%)	Mean (SD)	n (%)	*p* Value
**Age**	61.38 ± 18.54	-	60.12 ± 20.41	-	62.40 ± 16.93	-	0.464
**Hospitalization unit**	-		-		-		
Medical units		114 (75)		49 (71)		65 (78.3)	0.301
Surgery units		38 (25)		20 (29)		18 (21.7)	
**Admission days**	32.65 ± 35.47	-	31.43 ± 36.91	-	33.66 ± 34.42	-	0.701
**Barthel**	27.18 ± 28.95	-	24.66 ± 28.30	-	29.27 ± 29.48	-	0.334
**NPI-Q**	6.99 ± 5.92	-	9.00 ± 6.26	-	5.28 ± 5.05	-	<0.001 **

** *p* < 0.01. Continuous variables were analyzed by an Independent Samples *t*-test, and qualitative variables by Fisher’s Exact Test and Pearson Chi-Square. NPI-Q: Neuropsychiatric Inventory Questionnaire; SD: standard deviation.

**Table 3:** Crude and adjusted associations between the baseline variables of informal caregivers and the presence of insomnia symptoms.

		Outcome: Insomnia Symptoms			
		Crude OR		Adjusted OR	
Variables			95% CI		95% CI
Female caregivers		1.96	0.79–4.88	0.86	0.23–3.18
Caregiver age		0.98	0.96–1.01	0.97	0.93–1.02
Patient age		0.99	0.98–1.01	0.99	0.97–1.02
Education level	Medium	1.09	0.52–2.28	1.16	0.36–3.73
	High	3.68	1.43–9.43	5.50 *	1.34–22.63
Hospital stay (h)		1.00	0.99–1.01	1.01	0.99–1.02
No intake of hypnotics		0.53	0.27–1.05	0.53	0.20–1.45
Sleeping site	Home	0.56	0.24–1.35	0.65	0.12–3.65
	Both	0.96	0.44–2.09	0.85	0.22–3.31
ZBI		1.06 **	1.03–1.09	1.02	0.98–1.07
HADS Depression		1.21 **	1.12–1.32	1.01	0.87–1.16
HADS Anxiety		1.25 **	1.14–1.37	1.15 *	1.01–1.31
Barthel		0.99	0.98–1.01	1.00	0.98–1.01
NPI-Q		1.12 **	1.06–1.20	1.09	1.00–1.20
SSQ6 Satisfaction		0.75 *	0.57–0.97	0.79	0.55–1.13
CD-RISC		0.98 *	0.95–0.99	0.99	0.95–1.02

* *p* < 0.05; ** *p* < 0.01. A logistic regression model was used. Reference categories: male sex, low education level, hypnotics intake, sleeping at hospital. The remaining variables are continuous. CD-RISC: Connor–Davidson Resilience Scale; CI: confidence interval; HADS: Hospital Anxiety and Depression Scale; NPI-Q: Neuropsychiatric Inventory Questionnaire; OR: odds ratio; SSQ6: Social Support Questionnaire-Short Form; ZBI: Zarit Burden Interview.

## Data Availability

Data are available upon reasonable request.
